# Quantifying intergenerational differences in community public space perception: a multimodal Electroencephalography-eye tracking study

**DOI:** 10.7717/peerj.21126

**Published:** 2026-04-15

**Authors:** Wei Shang, Dexian Lu

**Affiliations:** 1 Hubei University of Technology, School of Civil Engineering and Architecture, Wuhan, Hubei, China; 2 Innovation Demonstration Base of Ecological Environment Geotechnical and Ecological Restoration of Rivers and Lakes, Wuhan, Hubei, China; 3 Hubei University of Technology, Key Laboratory of Intelligent Health Perception and Ecological Restoration of Rivers and Lakes, Wuhan, Hubei, China

**Keywords:** Intergenerational differences, EEG-eye tracking technology, Community public space

## Abstract

Against the dual backdrop of global aging and urbanization, the design of community public spaces for intergenerational integration has become a crucial approach to enhancing the quality of life for children and the elderly. However, existing studies mostly rely on subjective questionnaires and interviews, which struggle to reveal the potential intergenerational neurocognitive differences—particularly lacking quantitative analysis of the subconscious mechanisms in spatial perception between the elderly and children. This study aims to construct a multimodal evaluation system by integrating electroencephalography (EEG) and eye tracking technology to uncover the differences in neurobehavioral responses between the elderly and child groups in community public spaces. In this study, stratified random sampling was adopted to select community samples and collect spatial images. Based on computer vision (Segment Anything Model), spatial element identification and classification were conducted. Through controlled experiments, EEG and eye-tracking data of 40 participants (25 elderly and 15 children) were synchronously collected to reveal intergenerational differences in spatial cognition. The results show that the elderly and children exhibit significantly different “neuro-behavioral response patterns” in area typologies such as Community Service Areas and Leftover Areas. For instance, the elderly present a “high emotional arousal—high functional attention” pattern in Community Service Areas, while children demonstrate “high exploration—high dynamic attention” characteristics in Leftover Areas. These findings provide empirical evidence for the design of spaces for intergenerational integration from the neurocognitive perspective and offer scientific support for the refined and human-oriented construction strategies of community public spaces.

## Introduction

The world is currently undergoing two profound demographic transitions: population aging and urbanization (Andrews et al., 2018). According to the World Population Prospects 2024 report by the United Nations Department of Economic Social Affairs (2024), the global population aged 65 and above is projected to exceed that of individuals under 18 by the late 2070s. Currently, the proportion of the population aged 65 and above has risen from 6.8% in 2000 to approximately 14.3% in 2024; it is expected to reach 16.3% by 2050 and may surpass 21% in the second half of this century, signaling a transition into a “super-aged” society.To address these shifts, the “Age-Friendly Cities” model launched by the World Health Organization (WHO) promotes supportive urban communities that encourage “active aging” by optimizing opportunities for health, participation, and safety (Zaidi & Howse, 2017; Kalache & Gatti, 2003). Simultaneously,the developmental environment for children also faces severe challenges (UNICEF, 2016). The UN Convention on the Rights of the Child (UNCRC) highlights that children may suffer negative physical and mental health impacts due to increased housing density, reduced green spaces, and worsening pollution (UNICEF, 2012). By 2050, over 70% of children are projected to live in urban areas, complicating the realization of children’s rights (UNICEF, 2009).

Given these structural changes, the dual demand for age-friendly and child-friendly cities has become increasingly prominent. Promoting intergenerational interaction through urban design is now a core issue in urban planning (Fang et al., 2023). As the fundamental unit of urban activity, the quality of community public spaces is directly linked to resident well-being (Tönnies, 1887). Sociologically, a community is an organized group defined by geographical, spiritual, kinship, and labor relations. Ferdinand Tönnies distinguished “community” (Gemeinschaft) and “society” (Gesellschaft) as fundamental structural elements, noting that the conscious union of people constitutes the inherent potential of the group (Ongenae et al., 2014). Building upon this sociological foundation, “community public spaces” are defined in this study as the shared outdoor environments within residential neighborhoods—encompassing functional plazas, green spaces, playgrounds, and pedestrian networks—that serve as the primary physical carriers for these social interactions and daily activities (Chirapiwat, 1999).

High-quality community public spaces play a significant role in enhancing resident well-being, accumulating social capital, and strengthening community cohesion (Francis et al., 2012). However, with the prominence of the “dual-aging” trend in global demographics, the frequency and intensity of intergenerational conflicts in these spaces have been escalating (Nelischer & Loukaitou-Sideris, 2023). In urban public spaces, conflicts between the elderly and children are significantly influenced by age stereotypes (Solow, 2017). Children-only spaces, in particular, often become frequent sites for intergenerational disputes over usage rights (Wu, Siu & Zhang, 2023). While intergenerational integration is widely advocated as a strategy to mitigate these conflicts, its sustainability relies on fostering organic interaction between the elderly and other age groups (Urick et al., 2017). Currently, theoretical research on intergenerational issues remains largely concentrated in social psychology, mental health, and sociology, emphasizing interpersonal communication (Wooden, 2020). However, existing studies on physical community spaces tend to adopt a general population perspective, lacking specialized evaluation metrics and design standards tailored to the specific satisfaction and physiological needs of vulnerable groups like children and the elderly (Orazani, Reynolds & Osborne, 2023).

Traditional methods for evaluating public spaces, such as self-report questionnaires and interviews, rely heavily on participants’ memory retrieval and linguistic expression. However, these methods face significant validity challenges in intergenerational studies. For the elderly, age-related decline in working memory may affect the accuracy of retrospective reporting, while children often lack the vocabulary to articulate complex spatial feelings precisely (Li & Luximon, 2018). Furthermore, cognitive processing of spatial environments largely occurs at a subconscious level. Therefore, objective observation tools with high temporal precision are required to bypass reporting biases and capture instinctive neurobehavioral responses. Electroencephalography (EEG)-Eye Tracking Technology, by recording the oscillatory activities of specific EEG frequency bands (*α* waves, *β* waves) and visual fixation trajectories, provides the possibility for quantifying the potential intergenerational neurocognitive differences in spatial perception (Zheng, Dong & Lu, 2014).

To quantify these subconscious responses, electroencephalography (EEG) offers a direct window into cortical activity. EEG rhythms are closely related to cognitive functional states and are typically categorized into specific frequency bands: *δ* waves (0.5–4 Hz) primarily occur during deep sleep; *θ* waves (4–7 Hz) are associated with drowsiness and transitional states; and *γ* waves (>30 Hz) reflect higher-order cognitive processing (Ahn et al., 2016). However, in the context of active spatial perception, the primary bands of interest are *α* and *β* waves. *α* waves (8–13 Hz) are most prominent during physical and mental relaxation and are suppressed during focused attention or tension, whereas *β* waves (14–30 Hz) are involved in active thinking and attentional focus.Based on this theoretical framework, the power ratio of *β* to *α* waves (*β*/*α*) is widely used as a key neural metric for assessing cortical activation and cognitive load. A decrease in *α* power typically reflects the dissipation of a relaxed state, while an increase in *β* power indicates cognitive activation. Consequently, an increase in the *β*/*α* ratio is interpreted as elevated emotional arousal (Khosla, Khandnor & Chand, 2020). Unlike single-frequency bands, this ratio effectively normalizes individual differences in baseline power. Research in environmental behavior has confirmed that this ratio can sensitively reflect differences in neural responses to different visual scenes, making it a suitable measure for assessing reactions to environmental perception (Li et al., 2021).

Complementing EEG, eye-tracking technology captures visual attention allocation through two primary oculomotor behaviors: fixations (stabilizing gaze on an object) and saccades (rapid ballistic movements between fixations). In the context of spatial perception, the selection of metrics must align with the cognitive processes being investigated. Saccade-related metrics (*e.g.*, amplitude, velocity) are predominantly used to evaluate visual search efficiency, navigational wayfinding speed, or oculomotor mechanics (Poole & Ball, 2006). However, according to the “Eye-Mind Hypothesis” proposed by Just and Carpenter, there is a strong temporal link between the location of the gaze and the focus of cognitive processing (Just & Carpenter, 1976). Crucially, visual information uptake and cognitive decoding occur almost exclusively during fixations, whereas visual input is largely suppressed during saccades (Rayner, 1998). Previous research has demonstrated that specific eye movement patterns are intrinsic markers of psychological states. For instance, increased fixation density on specific environmental features typically reveals a subjective preference or heightened “visual interest”, while distinct scan paths can reflect the observer’s strategy for information gathering (Orquin & Loose, 2013). Since the primary objective of this study is to quantify the intensity of interest and the cognitive decoding depth of specific spatial elements (*e.g.*, distinguishing functional amenities from natural landscape), rather than the speed of visual search, fixation-based metrics are the most ecologically valid choice. Research indicates that fixation count and duration are positively correlated with the depth of information extraction and the semantic importance of the visual target (Henderson, 2007). Consequently, this study adopts fixation count and distribution (heatmaps) as the core metrics, excluding saccade and dwell-time metrics that are more indicative of search mechanics than the qualitative appraisal of static environmental aesthetics.

The in-depth integration of EEG signals and eye movement behavioral characteristic data has become the focus of attention for many researchers (Tahri Sqalli et al., 2023). However, its application in the field of architecture is still in its infancy. In the field of architecture, many scholars have used EEG technology or eye tracking technology to study architectural colors, architectural spaces, urban spaces, and other topics (Sun & Li, 2021; Erkan, 2018; Senkler et al., 2025). Nevertheless, research that combines EEG technology and eye tracking technology, focusing on the construction of community public spaces and intergenerational integration between the elderly and children, remains in a blank stage.

This study aims to fill the gap in the field of intergenerational space research within neuroarchitecture. By innovatively integrating computer vision (Segment Anything Model, SAM) with neuroergonomics (synchronized EEG-eye tracking), we constructed a multimodal evaluation system with second-level precision. This system automatically extracts outdoor community space elements and synchronously collects real-time neurocognitive data (*β*/*α* wave oscillations and gaze trajectories) from elderly and child groups. This approach breaks through the limitations of traditional subjective methods (such as satisfaction scales), allowing for the quantitative revelation of subconscious cognitive differences and conflicts between generations.

Ultimately, this study is committed to providing neurocognitive evidence-based design strategies, evaluation indicators, and decision-support tools for the scientific construction of community public spaces fostering intergenerational integration. In doing so, it aims to effectively alleviate intergenerational conflicts and enhance the spatial experience and sense of community belonging for different age groups.

## Materials & Methods

This study employed a between-group comparative experimental design to quantitatively reveal neurocognitive differences in intergenerational spatial perception by comparing the multimodal responses of the Elderly Group and the Child Group to standardized visual stimuli. All procedures were approved by the Research Ethics and Technology Safety Committee of Hubei University of Technology (Approval No: HBUT20250045).

### Study area and spatial sampling

The sampling frame comprised 30 initial communities in Wuhan. In this study, a “community” specifically refers to a “gated residential neighborhood” (known as Xiaoqu in China), which serves as the fundamental spatial unit of urban housing. These communities are characterized by distinct physical enclosure, shared property management, and exclusive communal public facilities used by residents. To enhance representativeness, communities were stratified into high, medium, and low levels based on the proportion of outdoor public space area. Using stratified random sampling, five communities were randomly selected from each stratum, resulting in 15 study samples.

**Figure 1:**
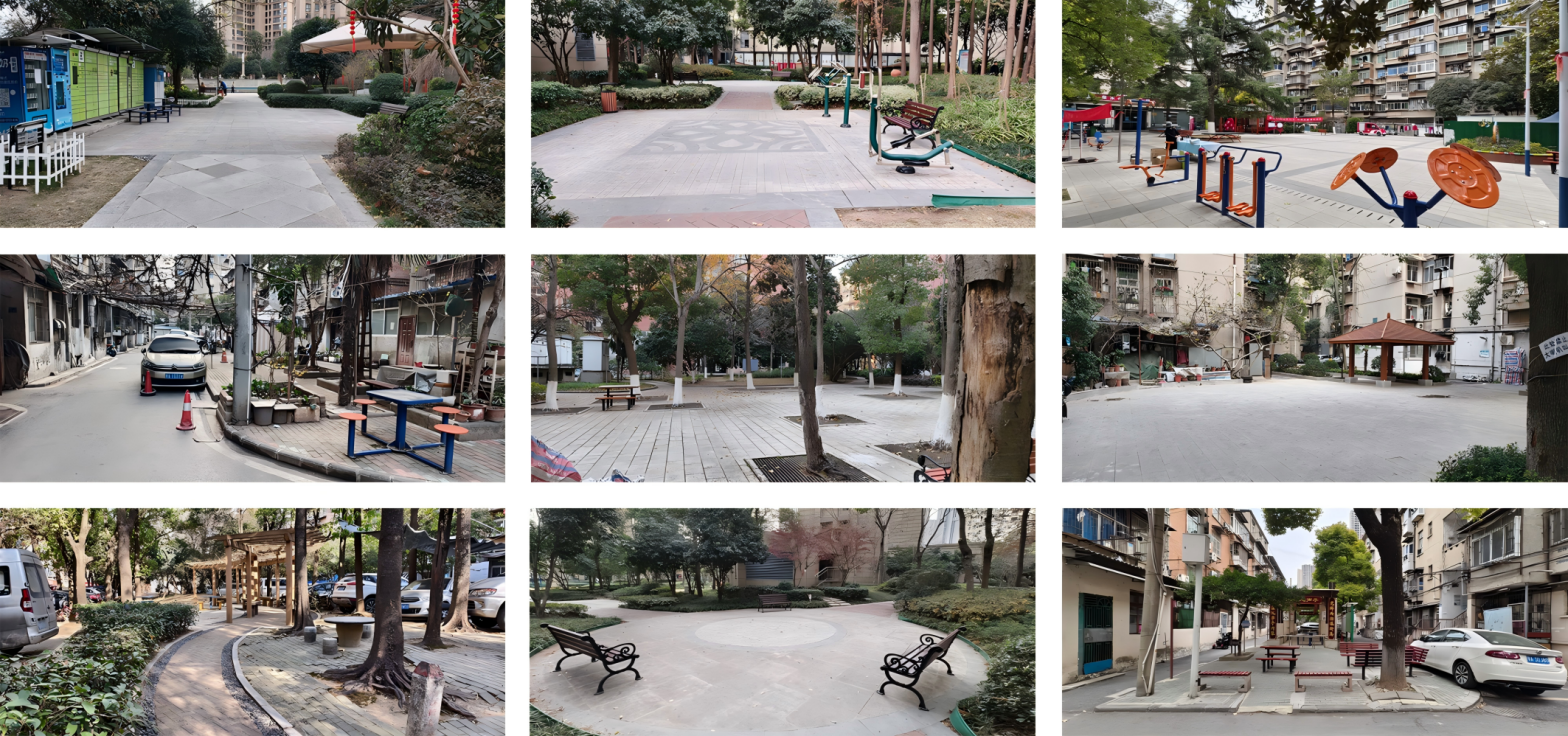
Examples of survey images.

Field data were collected from June 2024 to April 2025 *via* a standardized photography protocol. Using a Canon EOS R5 camera, high-resolution images were captured at a standardized human eye level (∼1.6 m) along key circulation paths and functional nodes to cover the representative spatial typologies. Image acquisition was scheduled on sunny days during peak activity hours (9:00–11:00 AM; 3:00–5:00 PM) to ensure authentic intergenerational dynamics and consistent lighting, yielding 246 images of outdoor Community Public Spaces (Fig. 1).

### Computer vision-based spatial classification

#### Semantic segmentation with Segment Anything Model (SAM)

**Figure 2:**
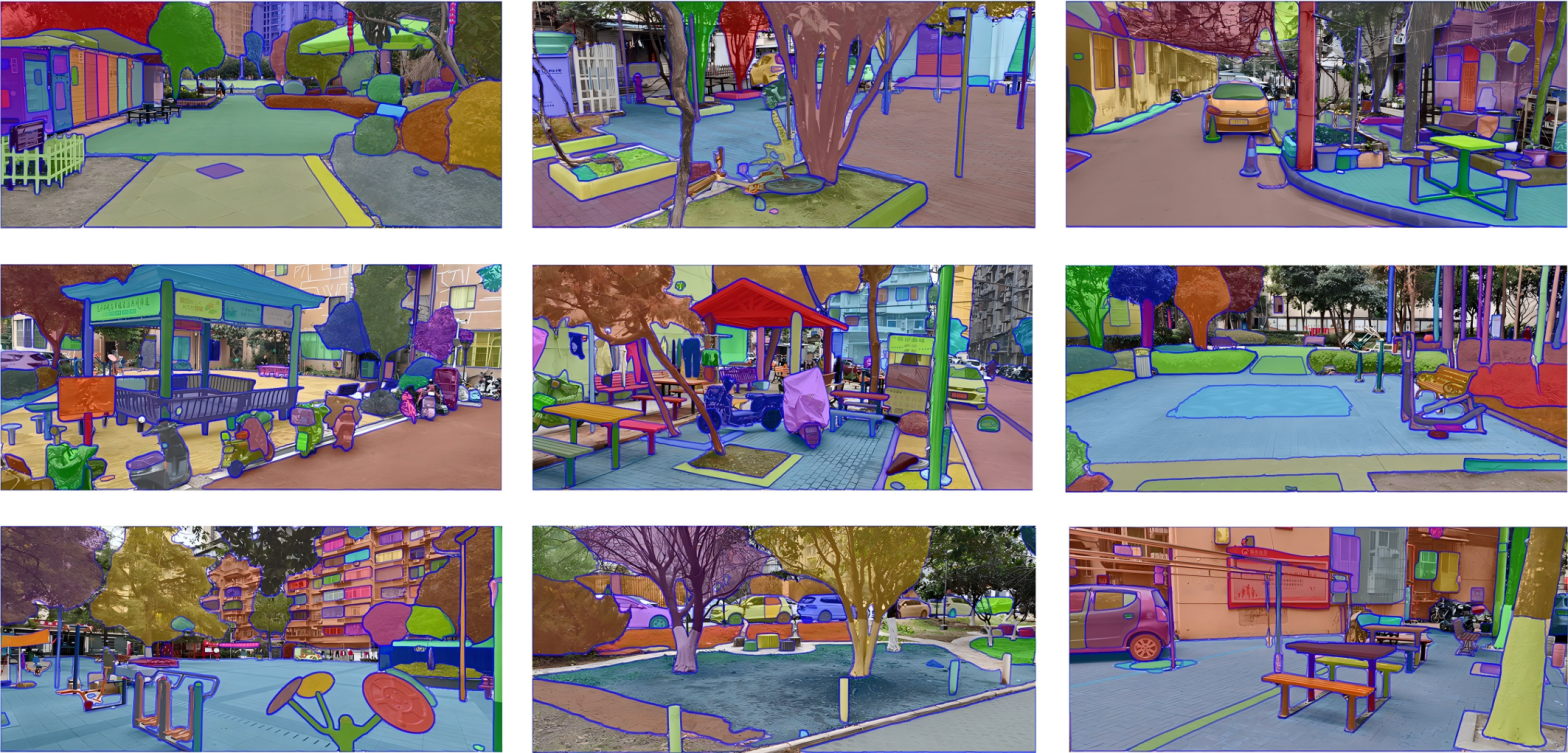
Results of image segmentation.

Semantic segmentation was performed on the 246 public space RGB images using the SAM-ViT-H model pre-trained on the SA-1B dataset (Zhang, Shen & Jiao, 2024) (Fig. 2). The segmentation results underwent a three-stage verification process comprising: automated system filtering, cross-verification by three independent annotators (inter-coder reliability: Fleiss’ Kappa = 0.81), and on-site validation of ambiguous elements. Ultimately, a total of 36 validated spatial elements were extracted. These elements were used to establish a four-category framework (Table 1). While this rigorous process ensured high accuracy, we acknowledge that certain dynamic elements (*e.g.*, Clothing, People) possess variable spatial locations compared to static infrastructure. To mitigate potential inconsistencies, these elements were annotated based on their strict pixel-level boundaries within the specific captured frames, independent of their temporary nature.

**Table 1:** Four-category framework of spatial elements.

Category	Element scope	Description
Natural ecology and ornamental elements	Trees, Shrubs, Ground Cover, Flower Beds, Potted Plants, Tree Wells	Tree Wells: Open soil areas specifically reserved around the base of trees within paved surfaces.
Infrastructure and structural elements	Roads, Pavement, Stairs, Electrical Boxes, Windows, Doors, Walls, Galleries, Utility Poles, Fences, Building Structures	Galleries: Covered walkways, corridors, or pergolas that provide shelter and connect different areas. Electrical Boxes: Utility cabinets housing electrical or telecommunication equipment visible in public areas.
Amenity and leisure facilities	Pavilions, Parcel Lockers, Fitness Equipment, Benches, Slides, Shades, Trash Cans, Vending Machines, Clothesline Poles, Canopies	Clothesline Poles: Vertical structures (often metal or bamboo) specifically installed to support lines for drying laundry. Parcel Lockers:Automated storage units used for the delivery and collection of packages. Pavilions: Open-sided roofed structures used for resting and social gatherings.
Dynamic use and management elements	Bicycles, People, Bollards, Game Tables, Banners, E-bikes, Clutter, Clothing, Cars	Clothing: Personal laundry hung in public spaces for drying, representing informal spatial occupation. bikes: Electric bicycles or scooters, distinguished from traditional bicycles due to higher speed and size. Clutter: Disorganized piles of personal items, debris, or discarded furniture occupying public space.

#### Functional area typology definition

To ensure the objectivity and reproducibility of the classification, quantitative criteria were established for functional area typology based on two specific dimensions: the Core Element Combination (*e.g.*, facility unit counts) and Spatial Morphological Characteristics (*e.g.*, visual area proportions) (Table 2).

**Table 2:** Quantitative definition standards for area typologies.

Area typology	Core element combination	Spatial morphological characteristics
Public activity areas	≥2 categories of Infrastructure and Structural Elements (dominated by Pavement) + ≥ 1 category of Amenity and Leisure Facilities	Visual area of hard pavement ≥ 40%
Community service areas	≥2 categories of Amenity and Leisure Facilities (dominated by Clothesline Poles and Parcel Lockers) + Dynamic Use and Management Elements	Unit count of daily auxiliary facilities (Parcel Lockers, Clothesline Poles, *etc.*) ≥ 3
Fitness and Recreation Areas	Amenity and Leisure Facilities (dominated by Fitness Equipment) + Infrastructure and Structural Elements	Unit count of Fitness Equipment ≥ 3
Social and Leisure Areas	Natural Ecology and Ornamental Elements + Amenity and Leisure Facilities (dominated by Benches and Pavilions)	Unit count of Benches + Pavilions ≥ 3; Coverage rate of natural elements >30%
Green Landscape Areas	Natural Ecology and Ornamental Elements	Coverage rate of non-natural elements <20%
Leftover Areas	Dynamic Use and Management Elements	No fixed functional elements
Multifunctional Areas	≥3 element categories with no single category dominant	Proportion of single-category elements <35%

#### Stratification of social interaction intensity

Based on the “spatial triad” (spatial practice-representations of space-representational spaces) proposed by Henri Lefebvre, the images of community public spaces were classified into Social Interaction Intensities: High-Intensity Social Interaction Areas, Low-Intensity Social Interaction Areas, and Latent Social Interaction Areas (Lefebvre, 2012).

### Stimulus presentation and equipment

#### Stimulus materials and presentation

Stimulus materials were selected through a rigorous classification and screening process of images depicting actual community spaces. The spatial typologies (categorized by spatial type and social interaction intensity) were derived from real-world observations and semantic segmentation, rather than a predetermined full factorial design. A systematic sampling strategy was employed to uniformly select two representative images for each category combination from a pool of 246 images, resulting in 30 experimental stimuli (resolution: 4, 096 × 1, 848 pixels). The total experimental session lasted approximately 6 min and 33 s, following a fixed sequence: a 5-second grey countdown screen, a 10-second static image stimulus, and a 3-second grey inter-stimulus interval. Stimuli were presented on a BenQ PD3220U 24-inch LCD monitor pre-calibrated to the sRGB color space with a stable brightness of 70 cd/m^2^.

#### Data acquisition hardware

EEG data were acquired using the SICHIRAY EEG Taurus sensor module, a single-channel system utilizing a dry-electrode interface. This device integrates the NeuroSky TGAM hardware module and its embedded e-Sense™ algorithm, which has been validated in prior environmental perception research (Rebolledo-Mendez et al., 2009). The configuration consists of three contact points: the active EEG electrode placed at the Fpz site (International 10-20 System) to monitor prefrontal activity, and the reference (Ref) and ground (GND) electrodes attached to the left earlobe *via* an integrated earclip. The module incorporates an analog front-end with a 50 Hz notch filter and digitizes raw EEG signals at a sampling rate of 512 Hz, providing outputs for raw signals, frequency power spectra, and derived cognitive indices.

Eye movements were recorded using a Tobii Eye Tracker 5, featuring a customized optical sensor with a sampling rate of 120 Hz designed for low-latency gaze capture. To ensure an unobstructed field of view, the eye tracker was mounted below the stimulus presentation monitor. A standardized 9-point calibration was performed for each participant, with data collection proceeding only if the average spatial error was less than 1.0°.

#### Software and synchronization

Data collection was managed through a synchronized multi-software protocol. Tobii Ghost software rendered real-time gaze trajectories and heatmaps overlaying the visual stimuli, while raw EEG data were simultaneously logged *via* SSCOM V5.13 (Serial Port Debugging Assistant). To ensure precise temporal alignment between the two modalities, a split-screen recording technique was implemented using OBS Studio (Open Broadcaster Software). The recording layout simultaneously captured the stimulus window (with Tobii Ghost visualization) and the real-time SSCOM data stream within a single composite video frame. This method guaranteed strict frame-by-frame synchronization between neurophysiological signals and visual attention events for subsequent analysis.

### Participants

The study recruited 40 participants, comprising 25 Elderly Group (age 60±5) and 15 children (age 14±3). Recruitment occurred from 3–17 May 2025. Exclusion criteria included history of neurological disorders, uncorrected visual impairment, or photosensitive epilepsy. Written informed consent was obtained from all participants (provided by guardians for children).

### Experimental procedure

**Figure 3:**
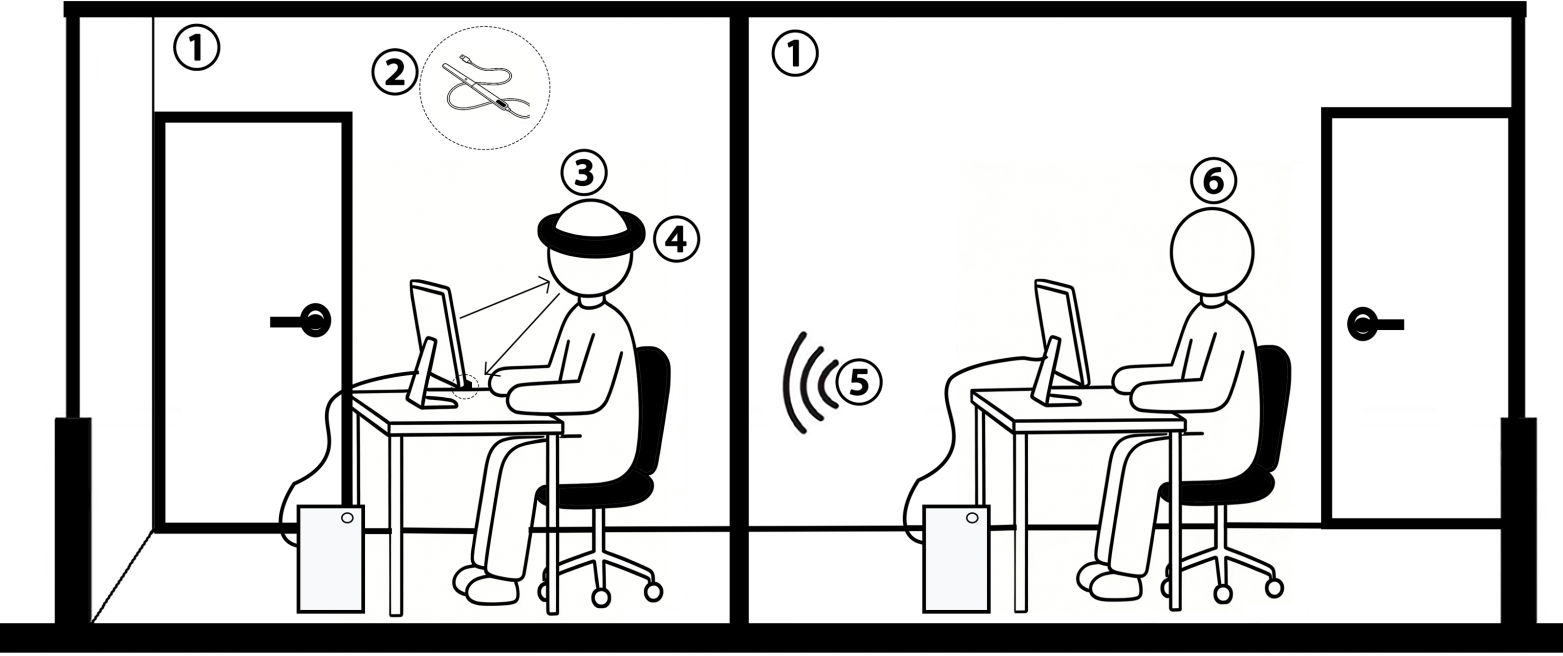
Experimental Schematic Diagram ① laboratory ② Tobii Eye Tracker 5 device ③ participant ④ SICHIRAY EEG Taurus sensor module ⑤ data recording ⑥ operator. Image credit: Adobe Photoshop.

The procedure consisted of three standardised phases:

(1) Preparation phase: After providing informed consent, participants were fitted with the EEG sensor, and signal quality was verified. Subsequently, a standard 9-point calibration was performed for the eye tracker to ensure gaze accuracy.

(2) Stimulus exposure phase: The 30 spatial images were presented in a pseudo-random order to prevent order effects. Participants were instructed to view the images freely and naturally without any specific task requirements.

(3) Data recording phase: Upon completion, timestamp-aligned raw EEG data (TXT format) and eye movement visualizations (MP4 format) were automatically archived for subsequent processing (Fig. 3).

### Data analysis and evaluation metrics

#### EEG data processing

The experiment utilized a single-channel SICHIRAY EEG Taurus sensor module integrated with the e-Sense™ algorithm for data acquisition. This algorithm enables real-time processing and output of raw brainwaves. While single-channel devices are less common in clinical diagnosis, their application in environmental psychology is well-established due to high ecological validity (Aspinall et al., 2015). Because the SICHIRAY EEG Taurus is based on the NeuroSky TGAM module and employs the same signal acquisition and processing framework, validation studies comparing the TGAM-based single-channel dry-electrode system against medical-grade wet-electrode arrays (*e.g.*, Neuroscan) have demonstrated strong correlations (*r* > 0.7) in measuring Alpha and Beta band activity, confirming their reliability for non-laboratory research (Rogers et al., 2016; Wyckoff et al., 2015).

In the preprocessing pipeline, the raw EEG signal (sampled at 512 Hz) first underwent a hardware-based 50 Hz notch filter to eliminate power line interference. Subsequently, Fast Fourier Transform (FFT) was employed to convert the time-domain signals into frequency-domain power spectra. The validity of this mathematical transformation for EEG feature extraction has been verified in multiple studies (Shaker, 2006). Consistent with the theoretical framework outlined in the Introduction, this study focused specifically on extracting the power spectra of Alpha (*α*, 8–13 Hz) and Beta (*β*, 14–30 Hz) bands. The emotional arousal level (I) was quantified using the standardized *β*/*α* ratio: I = B_*β*_/A_*α*_, where B_*β*_ and A_*α*_ represent the power spectral density of the beta and alpha bands, respectively. As established in environmental behavior research, this ratio effectively normalizes individual differences in baseline power and serves as a robust index for assessing cortical activation and cognitive load (Khosla, Khandnor & Chand, 2020; Li et al., 2021).

### Statistical analysis of EEG data

To quantitatively assess the intergenerational differences in emotional arousal (*β*/*α* ratio) during community public space perception, a Linear Mixed-Effects Model (LMM) was constructed using the “Mixed Models” module in IBM SPSS Statistics 27. The model included Group (Elderly *vs* Child), Area Typology, and Social Interaction Intensity as fixed effects, along with all possible two-way and three-way interactions among these factors. To account for individual differences in baseline physiological arousal, a random intercept was included for each participant (Subject ID). The Restricted Maximum Likelihood (REML) estimation method was employed.

Prior to the final analysis, the normality of the residuals was verified using Q-Q plots, which showed no significant deviations from a normal distribution. Regarding model selection, we also considered a model including “Image ID” as a crossed random effect. However, comparisons based on the Akaike Information Criterion (AIC) and Bayesian Information Criterion (BIC) indicated that the inclusion of image-level random effects did not significantly improve the model fit. Therefore, to maintain model parsimony, the final model retained only the random intercept for participants.

Crucially, regarding the unequal sample sizes between the elderly group (*n* = 25) and the child group (*n* = 15), the LMM framework was explicitly selected due to its inherent robustness in handling unbalanced datasets using Maximum Likelihood Estimation without listwise deletion. To ensure valid statistical inference, the Satterthwaite approximation was applied to estimate the denominator degrees of freedom, and Levene’s test was performed to confirm that the sample size disparity did not violate the assumption of homogeneity of variance.

### Eye tracking data processing

**Figure 4:**
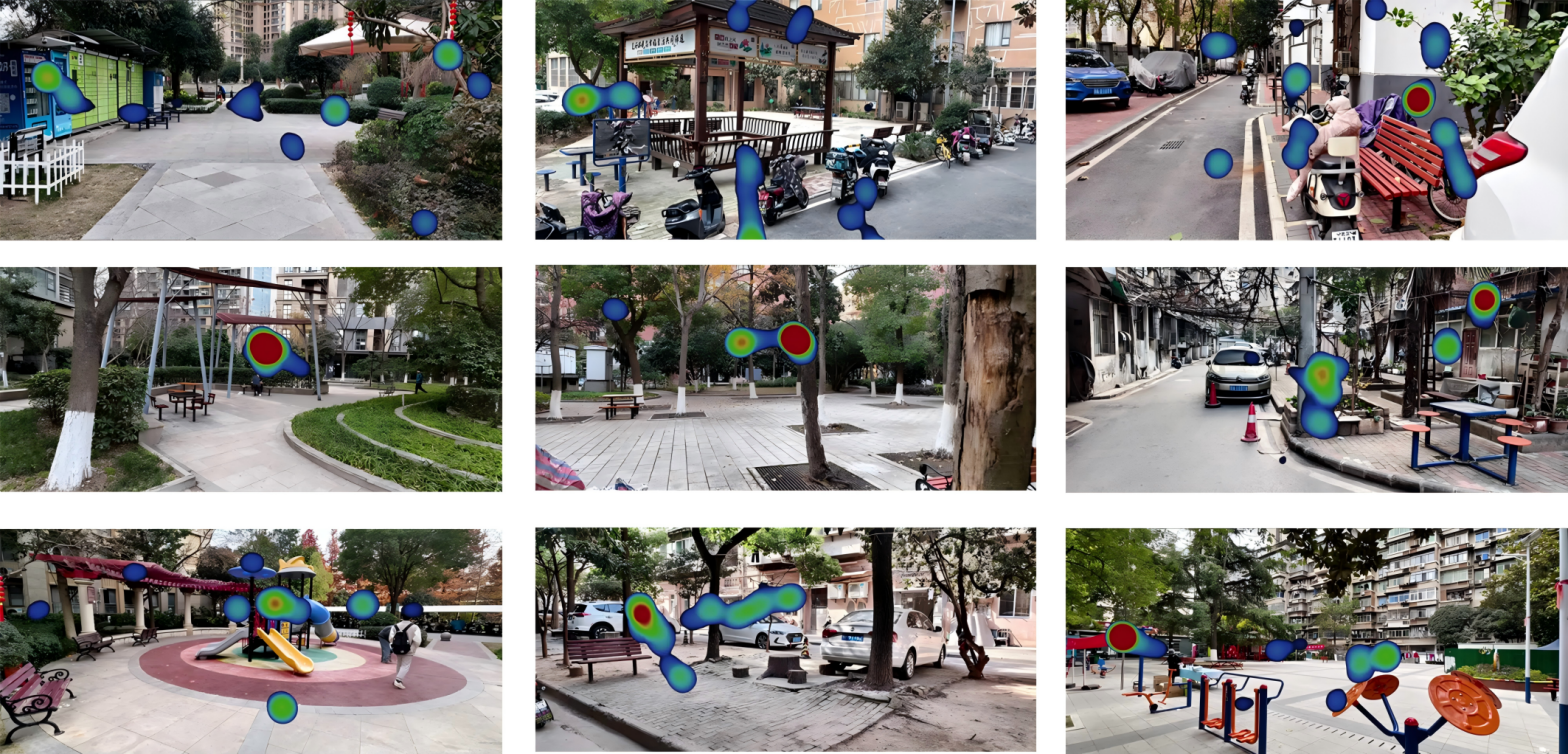
Eye tracking heatmaps.

Notes: SEA_m_: Attention proportion of the m-th spatial element category (%); C_m_: Total number of fixation points of participants on the m-th element category; ΣC_k_: Total number of fixation points of participants on all 36 element categories; m ∈[1,36]: Corresponding to the spatial element classification in Table 2 (*e.g.*, m =1: Trees, m =2: Shrubs, …, m =36: Cars).

Fixation count represents the total frequency of gaze stabilization on specific targets. A higher fixation count typically indicates elevated visual interest or greater information density within a region (Schirpke, Tasser & Lavdas, 2022). To visualize the spatial distribution of attention, eye movement videos from all 40 participants were processed using ffmpeg and segmented at a rate of one frame per second, generating 12,000 gaze heatmap frames (Fig. 4). A minimum duration threshold of 60 ms was utilized to classify ocular events as fixations.

To quantify visual attention towards specific spatial elements while controlling for variations in stimulus attractiveness and the imbalance in sample size (25 elderly *vs.* 15 children), a normalized metric termed the Spatial Element Attention (SEA) index was developed. This index calculates the relative proportion of fixations allocated to each of the 36 spatial element categories identified by the Segment Anything Model (SAM), ensuring comparability across groups and trials. The calculation formula is as follows: SEA_m_ = (C_m_/ΣC_k_) ×100%.

## Results

### EEG results

Box plots were used to illustrate the distribution characteristics of Emotional Arousal Levels. They present the central tendency and dispersion through medians, interquartile ranges (IQR), and outliers. To further quantify the relationships between these arousal levels and Area Typology or Social Interaction Intensity, and to test the statistical significance of observed differences, correlation analyses were subsequently conducted.

#### Analysis of intergenerational differences in emotional arousal

The results of the Linear Mixed Model (Type III Tests of Fixed Effects) (Table 3) and the covariance parameter estimates (Table 4) are presented. The analysis revealed that the main effect of Group was not significant (F(1, 39.041) = 0.103, *p* = 0.750).

**Table 3:** Fixed effects test of linear mixed model (dependent variable: *β*/*α* Ratio).

Source	Numerator degrees of freedom	Denominator degrees of freedom	F value	Significance (P)
Intercept	1	39.041	9,521.141	<0.001
Group	1	39.041	0.103	0.750
Area Typology	6	11,388.365	3.908	<0.001
Social Interaction Intensity	2	11,388.265	2.692	0.068
Group × Area Typology	6	11,388.365	2.156	0.044
Group × Social Interaction Intensity	2	11,388.265	2.144	0.117
Area Typology × Social Interaction Intensity	6	11,388.447	1.269	0.268
Group × Area Typology × Social Interaction Intensity	6	11,388.447	2.816	0.010

**Table 4:** Estimates of covariance parameters for the linear mixed model.

Parameter	Estimate	Standard Error	Wald Z	Significance (P)	95% Confidence Interval	
					Lower bound	Upper bound
Residual	0.182743	0.002422	75.459	<0.001	0.178057	0.187552
Intercept [Subject = ID]	0.005909	0.001502	3.933	<0.001	0.003590	0.009726

Regarding the random effects, the Wald Z test for the participant intercept was statistically significant (*Z* = 3.933, *p* < 0.001), confirming that accounting for individual baseline differences was necessary. The Intraclass Correlation Coefficient (ICC) calculated from the variance estimates (${\mathrm{sigma}}_{\mathrm{subj}}^{2}=0.006$, ${\mathrm{sigma}}_{\mathrm{resid}}^{2}=0.183$) was 0.031. This indicates that while individual physiological baselines account for approximately 3.1% of the total variance, the majority of the variability is driven by environmental factors and interactions.

Crucially, the analysis yielded statistically significant interaction effects: a two-way interaction between Group and Area Typology (*p* = 0.044) and a three-way interaction (*p* = 0.010). These results indicate that the pattern of emotional arousal across space types differs significantly between generations.

#### Elderly Group’s EEG results

**Figure 5:**
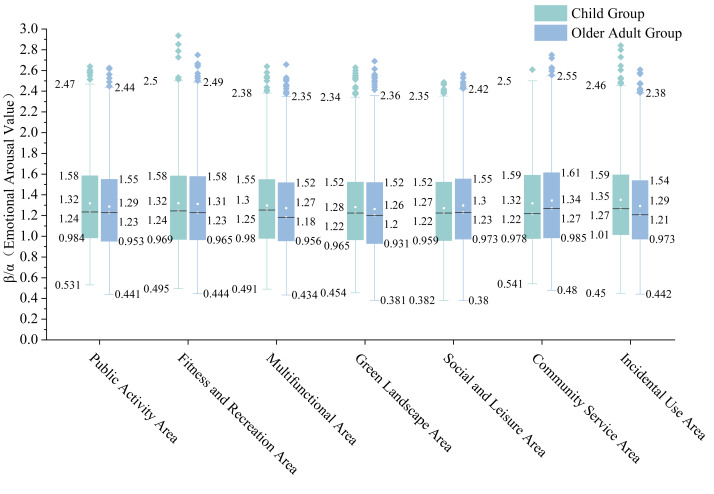
Comparative box plots of emotional arousal levels by area typology between the Child Group and the Elderly Group.

Analysis by Area type (Fig. 5) showed the Community Service Area recorded the highest median Emotional Arousal Value (1.27), significantly exceeding other Areas (range: 1.18−1.23). The Multifunctional Areas showed the lowest median (1.18). Regarding dispersion, the Community Service Areas demonstrated the largest IQR (0.625), while the Multifunctional Areas showed the smallest (0.564). All Area types contained high-end outliers, with the Green Landscape Areas and Multifunctional Areas containing the highest numbers.

**Figure 6:**
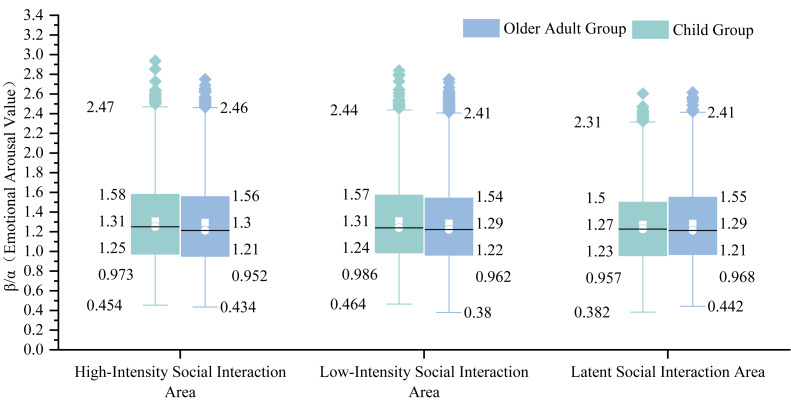
Comparative box plots of emotional arousal levels in social interaction intensity areas between the Child Group and the Elderly Group.

Analysis by Social Interaction Intensity Areas (Fig. 6) revealed the Low-Intensity Social Interaction Areas had a slightly higher median Emotional Arousal Value (1.22) than both the High-Intensity and Latent Social Interaction Areas (both 1.21). The High-Intensity Social Interaction Areas exhibited the largest IQR (0.608), compared to 0.578 and 0.582 for the Low-Intensity and Latent Social Interaction Areas respectively. The Low-Intensity Social Interaction Areas contained the most outliers.

#### EEG results for Child Group

Analysis by Area type (Fig. 5) indicated the Leftover Areas had the highest median Emotional Arousal Value (1.27), while the Green Landscape Areas, Social and Leisure Areas, and Community Service Areas shared the lowest median (1.22). The Fitness and Recreation Areas and Community Service Areas showed larger IQRs (0.611 and 0.612 respectively), while the Green Landscape Areas had the smallest (0.555). The Multifunctional Areas and Green Landscape Areas contained the most outliers.

Analysis by Social Interaction Intensity Areas (Fig. 6) showed median values of 1.25, 1.24, and 1.23 for the High-Intensity, Low-Intensity, and Latent Social Interaction Areas respectively. The High-Intensity Social Interaction Areas had the largest IQR (0.607), while the Latent Social Interaction Areas had the smallest (0.543). The Low-Intensity Social Interaction Areas contained the most outliers.

#### Intergroup EEG differences

Comparison by Area type (Fig. 5) revealed that the Child Group exhibited higher median Emotional Arousal Values than the Elderly Group in all Areas except the Social and Leisure Areas and Community Service Areas. The Child Group’s medians in the Multifunctional Areas and Leftover Areas (1.25, 1.27) were significantly higher than those of the Elderly Group, while the Elderly Group’s median in the Community Service Areas (1.27) was significantly higher. The Elderly Group showed larger IQRs than the Child Group in all Area types except the Multifunctional and Leftover Areas.These observed descriptive differences were statistically validated by the significant “Group × Area Typology” interaction in the Linear Mixed Model (F(6, 11388.365) = 2.156, *p* = 0.044), confirming that the pattern of emotional arousal across community spaces is fundamentally different between generations.

Comparison by Social Interaction Intensity Areas (Fig. 6) indicated that the Child Group had higher median Emotional Arousal Values across all three levels. The Elderly Group demonstrated a significantly larger IQR in the Latent Social Interaction Areas (0.582) compared to the Child Group (0.543). The Child Group’s outliers in the High-Intensity Social Interaction Areas showed greater volatility, whereas the Elderly Group’s outliers in the Low-Intensity Social Interaction Areas were more numerous and densely distributed.Furthermore, the complex interplay between generation, spatial function, and social context was confirmed by the significant three-way interaction (“Group × Area Typology × Social Interaction Intensity”) in the LMM (F(6, 11388.447) = 2.816, *p* = 0.010), indicating that intergenerational differences in arousal are dynamically modulated by the social intensity of the environment.

### Eye tracking results

Analysis through the Spatial Element Attention (SEA) index revealed distinct characteristics and differences in visual attention allocation between the Elderly Group and Child Group. Heatmap matrices illustrated the distribution of SEA values across spatial elements under varying environmental conditions, while dual-line charts facilitated intergroup comparisons.

#### Eye tracking results for the Elderly Group

**Figure 7:**
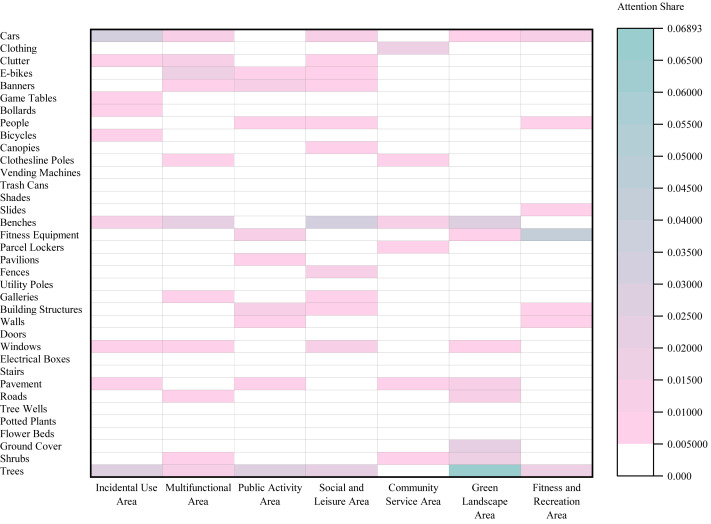
Heatmap of Spatial Element Attention (SEA) values across different area typologies in the Elderly Group.

Analysis of Area Typology (Fig. 7) revealed that Dynamic Use and Management Elements attracted the most attention in both Leftover Areas (SEA = 0.0653; peak element: ‘Cars’, 0.0328) and Multifunctional Areas (SEA = 0.0495; peak: ‘Benches’, 0.0209). In contrast, Green Landscape Areas were dominated by Natural Ecology and Ornamental Elements (SEA = 0.1069; peak: ‘Trees’, 0.0662), while Community Service Areas prioritized Amenity and Leisure Facilities (SEA = 0.0291; peak: ‘Clothing’, 0.0182).

**Figure 8:**
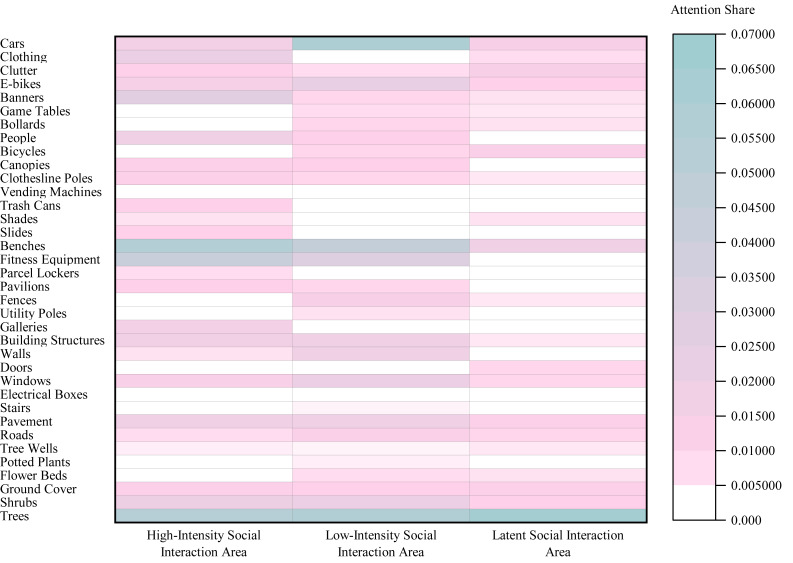
Heatmap of Spatial Element Attention (SEA) values across different social interaction intensity areas in the Elderly Group.

Regarding Social Interaction Intensity (Fig. 8), the highest SEA values shifted across categories: Amenity and Leisure Facilities led in High-Intensity Social Interaction Areas (0.143), Dynamic Use and Management Elements in Low-Intensity Social Interaction Areas (0.1163), and Natural Ecology and Ornamental Elements in Latent Social Interaction Areas (0.0905).

#### Eye tracking results for the Child Group

**Figure 9:**
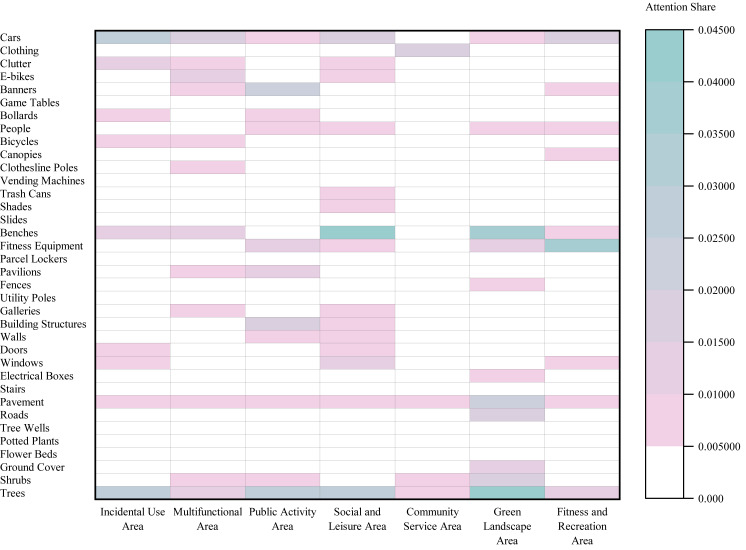
Heatmap of Spatial Element Attention (SEA) values across different area typologies in the Child Group.

Analysis of Area Typology (Fig. 9) indicated that Dynamic Use and Management Elements attracted the most attention in both Leftover Areas (SEA = 0.0578; peak element: ‘Cars’, 0.0289) and Multifunctional Areas (SEA = 0.0599). In Green Landscape Areas, attention focused on Natural Ecology and Ornamental Elements (SEA = 0.0726; peak: ‘Trees’, 0.0421), while Fitness and Recreation Areas prioritized Amenity and Leisure Facilities (SEA = 0.0467; peak: ‘Fitness Equipment’, 0.0434).

**Figure 10:**
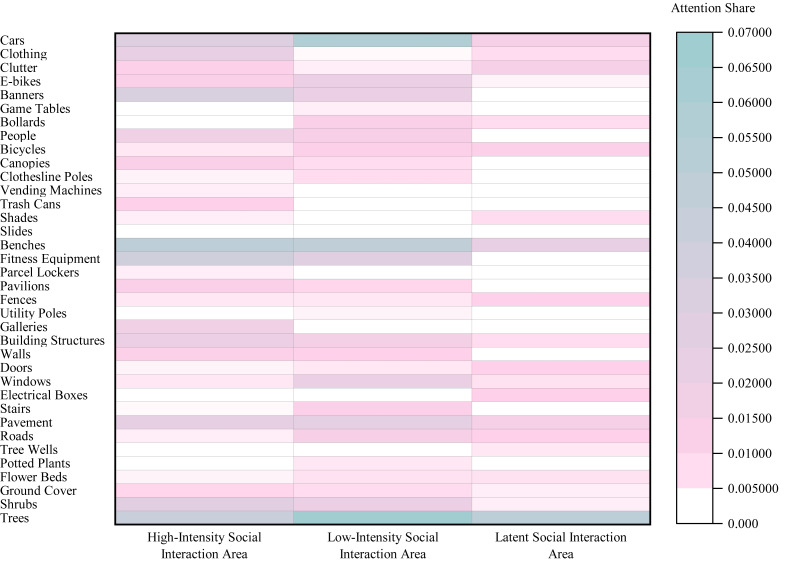
Heatmap of Spatial Element Attention (SEA) values across different social interaction intensity areas in the Child Group.

Regarding Social Interaction Intensity (Fig. 10), the highest SEA values shifted across categories: Amenity and Leisure Facilities led in High-Intensity Social Interaction Areas (0.1238), Dynamic Use and Management Elements in Low-Intensity Social Interaction Areas (0.1324), and Natural Ecology and Ornamental Elements in Latent Social Interaction Areas (0.0619).

#### Intergroup differences

**Figure 11:**
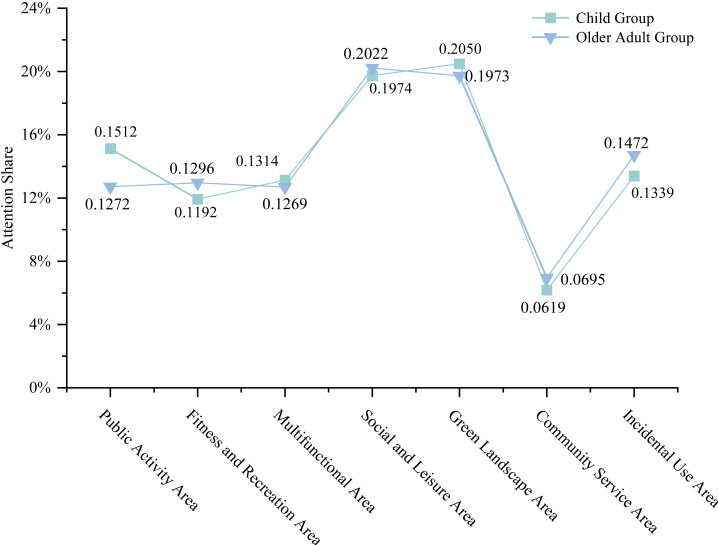
Comparative double line chart of SEA values by area typology between the Elderly Group and the Child Group.

Comparison by Area type (Fig. 11) showed that the Child Group had a significantly higher overall SEA in Public Activity Areas (0.1512) than the Elderly Group (0.1272), whereas the Elderly Group exhibited significantly higher SEA in Fitness and Recreation Areas (0.1296) and Leftover Areas (0.1472).

**Figure 12:**
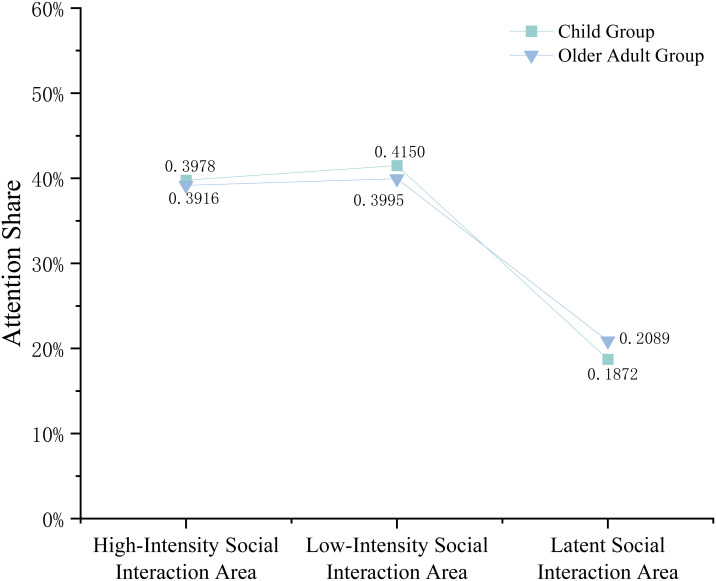
Comparative double line chart of SEA values in social interaction intensity areas between the Elderly Group and the Child Group.

Analysis by Social Interaction Intensity Areas (Fig. 12) indicated that the Child Group had a significantly higher SEA in Low-Intensity Social Interaction Areas (0.4150) compared to the Elderly Group (0.3995), while the Elderly Group demonstrated a significantly higher SEA in Latent Social Interaction Areas (0.2089).

### EEG-eye tracking data fusion

This chapter presents the cross-modal alignment and fusion of EEG emotional arousal values (*β*/*α*) and eye-tracking spatial element attention proportion values (SEA). This integration aims to objectively reveal the correlations between neurophysiological responses and visual behaviors in the Elderly Group and the Child Group.

#### EEG-eye tracking data fusion for the Elderly Group

The integration of multimodal data for the Elderly Group reveals distinct neuro-behavioral correlations across different spatial typologies (Table 5).

**Table 5:** Comparison of EEG-eye-tracking data for the Elderly Group across different area typologies.

Area typology	Median	IQR	Outlier characteristics	Spatial element type with the highest SEA value	Main focused elements
Public activity areas	1.23	0.597	High arousal end	Dynamic use and management category	Advertising signs, electric vehicles, people, *etc.*
Fitness and recreation areas	1.23	0.615	High arousal end	Service Facilities and Leisure Category	Sports equipment, cars, people, *etc.*
Multifunctional areas	1.18	0.564	High arousal end	Dynamic use and management category	Cars, debris, advertising signs, *etc.*
Social and leisure areas	1.23	0.577	High arousal end	Infrastructure and component category	Cars, debris, advertising signs, *etc.*
Green landscape areas	1.20	0.589	High arousal end with the largest quantity	Natural ecology and ornamental category	Arbor trees
Community service areas	1.27	0.625	High arousal end with the smallest quantity	Service facilities and leisure category	Clothes, clothes drying racks, express lockers, *etc.*
Leftover areas	1.21	0.567	High arousal end	Dynamic use and management category	Cars, debris, chess tables, *etc.*

In Community Service Areas, the elderly exhibited the highest median Emotional Arousal (1.27) and the widest variation (IQR = 0.625), with minimal outliers. This elevated arousal coincides with peak visual attention toward Amenity and Leisure Facilities (SEA = 0.0291), specifically focused on utilitarian elements such as “Clothing” (SEA = 0.0182), “Drying Racks”, and “Express Lockers”.

Conversely, Multifunctional Areas elicited the lowest median arousal (1.18) and the most constrained variation (IQR = 0.564), with outliers exclusively in the high-arousal range. Visually, attention was primarily allocated to Dynamic Use and Management Elements (SEA = 0.0495), distributed across elements such as “Cars”, “Debris”, and “Advertising Signs”.

In Green Landscape Areas, the arousal data showed significant volatility (wide IQR) and the highest density of outliers. This physiological response parallels a dominant visual focus on Natural Ecology and Ornamental Elements (SEA = 0.1069), with “Trees” (SEA = 0.0662) serving as the primary fixation target.

Lastly, Leftover Areas were characterized by relatively low arousal (1.22). Eye-tracking data indicates this state is associated with attention to Dynamic Use and Management Elements (SEA = 0.0653), particularly specific objects including “Cars”, “Debris”, and “Chess Tables”.

Furthermore, the fused data elucidates the correlation between arousal and attention across different levels of social interaction intensity (Table 6). In Low-Intensity Social Interaction Areas, the elderly exhibited the highest median Emotional Arousal (1.22) alongside the highest frequency of outliers. This heightened physiological response aligns with a visual dominance of Dynamic Use and Management Elements (SEA = 0.1163) within these spaces. Conversely, Latent Social Interaction Areas were characterized by the most stable emotional response (narrowest IQR) and minimal outliers. Visual attention in these environments was primarily directed toward Natural Ecology and Ornamental Elements (SEA = 0.0905), whereas Amenity and Leisure Facilities received the least attention (SEA = 0.0243).

**Table 6:** Comparison of EEG-eye-tracking data for the Elderly Group across different social interaction intensity areas.

Social interaction intensity areas	Median	IQR	Outlier characteristics	Spatial element type with the highest SEA value	Main focused elements
High-intensity social interaction areas	1.21	0.608	High arousal end	Amenity and leisure facilities	Benches, Fitness Equipment, Trees
Low-intensity social interaction areas	1.22	0.578	High arousal end with the largest quantity	Dynamic use and management elements	Cars, Benches, Trees
Latent social interaction areas	1.21	0.582	High arousal end with the smallest quantity	Natural ecology and ornamental elements	Trees

#### EEG-eye tracking data fusion for the Child Group

The integration of multimodal data for the Child Group reveals distinct correlations between Emotional Arousal Values and visual attention across different spatial typologies (Table 7).

**Table 7:** Comparison of EEG-eye tracking data for the Child Group across different area typologies.

Area typology	Median	IQR	Outlier characteristics	Spatial element type with the highest SEA value	Main focused elements
Public activity areas	1.24	0.596	High arousal end	Dynamic use and management elements	Banners, Cars, People, *etc.*
Fitness and recreation areas	1.24	0.611	High volatility and high arousal end	Amenity and leisure facilities	Fitness Equipment, Cars, People, *etc.*
Multifunctional areas	1.25	0.570	High arousal end with the second-largest quantity	Dynamic use and management elements	Cars, Clutter, Banners, *etc.*
Social and leisure areas	1.22	0.561	High arousal end	Amenity and leisure facilities	Cars, Clutter, Benches, *etc.*
Green landscape areas	1.22	0.555	High arousal end with the largest quantity	Natural ecology and ornamental elements	Trees, Cars, Benches, *etc.*
Community service areas	1.22	0.612	High arousal end with the smallest quantity	Dynamic use and management elements	Clothing, Pavement, Trees, *etc.*
Leftover areas	1.27	0.580	Most volatile and higher values	Dynamic use and management elements	Cars, Clutter, Bollards, *etc.*

The highest median Emotional Arousal Value (1.27) was recorded in Leftover Areas, where outliers exhibited the most significant volatility and higher values. This intense physiological response coincides with the highest SEA value for Dynamic Use and Management Elements (0.0578), with visual attention sharply focused on specific objects such as “Cars”, “Bollards”, and “Bicycles”.

In Fitness and Recreation Areas, a relatively high median arousal (1.24) was observed alongside the second-widest IQR (0.611), indicating a high degree of data dispersion. Eye-tracking data confirmed that visual attention here was concentrated on Amenity and Leisure Facilities (SEA = 0.0467), particularly “Fitness Equipment” (SEA = 0.0434).

The lowest median Emotional Arousal Value (1.22) was shared by Green Landscape Areas and Community Service Areas. However, their visual attention patterns diverged significantly. In Green Landscape Areas, the Child Group demonstrated the highest SEA value (0.0726) for Natural Ecology and Ornamental Elements, focusing primarily on “Trees” (SEA = 0.0421). In contrast, in Community Service Areas—which contained the fewest outliers—attention was directed toward Dynamic Use and Management Elements (SEA = 0.0203), specifically elements such as “Clothing” (SEA = 0.0167).

Lastly, Multifunctional Areas elicited a relatively high median arousal (1.25) and the second-highest frequency of outliers. Correspondingly, the SEA value for Dynamic Use and Management Elements was very high (0.0599), reflecting an extensive visual scanning strategy covering diverse elements including “Cars”, “Clutter”, “Banners”, and “E-bikes”.

The neural and behavioral responses of the Child Group also exhibit specific correlations across differing levels of social interaction intensity (Table 8).

**Table 8:** Comparison of EEG-eye tracking data for the Child Group across different social interaction intensity areas.

Social interaction intensity areas	Median	IQR	Outlier characteristics	Spatial element type with the highest SEA value	Main focused elements
High-intensity social interaction areas	1.25	0.607	High volatility and higher values	Amenity and leisure facilities	Benches, Fitness Equipment, Trees, *etc.*
Low-intensity social interaction areas	1.24	0.584	High arousal end with the largest quantity	Dynamic use and management elements	Cars, Benches, Trees
Latent social interaction areas	1.23	0.543	High arousal end	Natural ecology and ornamental elements	Trees

In High-Intensity Social Interaction Areas, the highest median Emotional Arousal Value (1.25) and the widest Interquartile Range (IQR = 0.607) were observed, with outliers demonstrating significant volatility and higher values. Eye-tracking data indicates that within these spaces, the SEA value for Amenity and Leisure Facilities was the highest (0.1238), with attention concentrated on elements such as “Benches” (SEA = 0.0472), “Fitness Equipment” (SEA = 0.0365), and “Banners” (SEA = 0.0315).

In Low-Intensity Social Interaction Areas, the median Emotional Arousal Value was slightly lower (1.24), yet this classification contained the highest frequency of outliers. Corresponding eye-tracking data shows that Dynamic Use and Management Elements attracted the most attention (SEA = 0.1324) in these spaces, with particular focus on “Cars” (SEA = 0.0558) and “Trees” (SEA = 0.0649).

Lastly, Latent Social Interaction Areas recorded the lowest median Emotional Arousal Value (1.23) and the narrowest IQR (0.543), indicating the most stable emotional responses. Visual data reveals that in these environments, the SEA value for Natural Ecology and Ornamental Elements was the highest (0.0619), with attention predominantly focused on “Trees” (SEA = 0.0477).

#### Difference analysis of EEG-eye tracking data fusion between the Elderly Group and the Child Group

Through cross-modal integration and cross-group comparison of EEG Emotional Arousal Values and Eye-tracking Visual Attention Data, this study identifies systematic differences in “Neuro-Behavioral Response Patterns” between the Elderly Group and the Child Group in the perception of community public space (Table 9).

**Table 9:** Comparison of “Neuro-Behavioral Response Patterns” between the Elderly Group and the Child Group across different area typologies.

Area typology	Group	Pattern characteristics
Community service areas	The Elderly Group	High emotional arousal-high attention to amenity and leisure facilities
	The Child Group	Low emotional arousal-low attention to amenity and leisure facilities
Leftover area	The Elderly Group	High emotional arousal-high attention to dynamic use and management elements
	The Child Group	Low emotional arousal-high attention to dynamic use and management elements
Multifunctional area	The Elderly Group	High emotional arousal-high attention to dynamic use and management elements
	The Child Group	Low emotional arousal (the lowest among all areas)-high attention to dynamic use and management elements
Green landscape area	The Elderly Group	Large variation in emotional response (larger IQR)-high natural ecology and ornamental elements
	The Child Group	Stable emotional response-high natural ecology and ornamental elements

In Community Service Areas, the Elderly Group exhibits a coupled pattern of “High Emotional Arousal (Median = 1.27)—High Attention to Amenity and Leisure Facilities (SEA = 0.0291)”. Both the median arousal value and outlier characteristics (the fewest in quantity) indicate a consistently high arousal response. In contrast, the Child Group presents a pattern of “Low Emotional Arousal (Median = 1.22)—Low Attention to Amenity and Leisure Facilities (SEA = 0.0203)” in this area.

In Leftover Areas, the two groups show significant intergenerational differentiation. The Child Group demonstrates a typical coupled pattern of “High Emotional Arousal (Median = 1.27)—High Attention to Dynamic Use and Management Elements (SEA = 0.0578)”, with outliers showing significant volatility. However, the Elderly Group presents a decoupled pattern of “Low Emotional Arousal (Median = 1.22)—High Attention to Dynamic Use and Management Elements (SEA = 0.0653)”, implying that high visual attention is not accompanied by corresponding positive emotional arousal.

In Multifunctional Areas, both groups show high visual attention to Dynamic Use and Management Elements (SEA = 0.0495 for the Elderly Group; SEA = 0.0599 for the Child Group). Nevertheless, their accompanying emotional arousal levels are distinctly different: the Child Group maintains a relatively high arousal level (Median = 1.25) with the highest frequency of outliers, whereas the Elderly Group records the lowest arousal level among all areas (Median = 1.18) with the narrowest IQR.

In Green Landscape Areas, both groups invest the highest level of visual attention in Natural Ecology and Ornamental Elements (SEA = 0.1069 for the Elderly Group; SEA = 0.0726 for the Child Group). However, the physiological response diverges: the IQR of the Elderly Group’s Emotional Arousal Values is relatively large with the highest frequency of outliers, while the Child Group’s emotional arousal response is notably more stable (IQR = 0.555).

Similarly, significant intergenerational differences in “Neuro-Behavioral Response Patterns” were observed across different Social Interaction Intensity Areas (Table 10).

**Table 10:** Comparison of “Neuro-Behavioral Response Patterns” between the Elderly Group and the Child Group across different social interaction intensity areas.

Social interaction intensity areas	Group	Pattern characteristics
High-intensity social interaction area	The Elderly Group	Low emotional arousal-high attention to amenity and leisure facilities
	The Child Group	High emotional arousal-high attention to amenity and leisure facilities
Low-intensity social interaction area	The Elderly Group	Low emotional arousal-high attention to dynamic use and management elements
	The Child Group	High emotional arousal-high attention to dynamic use and management elements
Latent social interaction area	The Elderly Group	Low emotional arousal-extremely high attention to natural ecology and ornamental elements
	The Child Group	Low emotional arousal-high attention to natural ecology and ornamental elements

In the High-Intensity Social Interaction Areas, the Child Group exhibits a synergistic pattern of “High Emotional Arousal (Median = 1.25)—High Attention to Amenity and Leisure Facilities (SEA = 0.1238)”, with attention highly concentrated on specific elements such as “Benches” and “Fitness Equipment”. Conversely, although the Elderly Group also directs substantial attention to Amenity and Leisure Facilities in these areas (SEA = 0.143), their emotional arousal level is relatively lower (Median = 1.21) and exhibits greater dispersion (IQR = 0.608).

The Low-Intensity Social Interaction Areas trigger contrastive patterns between generations. The Elderly Group is characterized by the highest frequency of outliers and high attention to Dynamic Use and Management Elements (SEA = 0.1163). In contrast, the Child Group presents a more intense engagement pattern, characterized by high emotional arousal (Median = 1.24), superior attention to Dynamic Use and Management Elements (SEA = 0.1324), and the highest frequency of outliers.

In the Latent Social Interaction Areas, the Child Group demonstrates a stable pattern of “Low Emotional Arousal (Median = 1.23)—High Attention to Natural Ecology and Ornamental Elements (SEA = 0.0619)”. Their emotional response is the most stable among all categories (narrowest IQR = 0.543), with attention focused on elements such as “Trees”. Compared to the Child Group, the Elderly Group exhibits a pattern of “Moderate Emotional Arousal (Median = 1.21)—Extremely High Attention to Natural Ecology and Ornamental Elements (SEA = 0.0905)”. While their visual investment in nature far exceeds that of the Child Group, the stability of their emotional response (IQR = 0.582) is comparatively lower.

## Discussion

By synchronously capturing EEG and Eye-tracking data, this study provides the first quantitative, multimodal evidence of the distinct neurocognitive disparities between the elderly and children in community public spaces. Validated by robust Linear Mixed Model analysis, the results confirm that the neurophysiological linkage between emotional arousal and functional space is fundamentally generation-specific. Furthermore, the significant three-way interaction involving Social Interaction Intensity identifies social context as a critical moderator, shaping how each generation neurologically engages with spatial environments. These findings validate behavioral preferences at the implicit neurocognitive level, offering a scientific basis for refined, intergenerational spatial design.

### Intergenerational differences in neuro-behavioral patterns of spatial perception

The core finding of this study is the revelation of distinctly different “Neuro-Behavioral Response Patterns” between the Elderly Group and the Child Group in specific area typologies. In the Community Service Areas, the Elderly Group exhibits a coupled pattern of “High Emotional Arousal-High Attention to Amenity and Leisure Facilities”, while the Child Group presents a pattern of “Low Emotional Arousal-Low Attention to Amenity and Leisure Facilities”. This finding aligns with Lawton’s Environmental Competence Theory, indicating that the elderly’s strong dependence on functional spaces reflects their need to maintain daily living abilities and independence (Howell, 1980). In contrast, children’s lack of interest in these practical functional elements may stem from their core need for exploration and play during development (Kyttä, 2004).

The intergenerational differentiation observed in the Leftover Areas is particularly significant. The Child Group’s pattern of “High Emotional Arousal-High Attention to Dynamic Use and Management Elements” supports Kyttä’s Child-Friendly Environment Theory, which states that unstructured and highly variable spaces are more likely to stimulate children’s exploratory behavior and creative play (Heft, 1988). Conversely, the Elderly Group’s decoupled pattern of “Low Emotional Arousal-High Attention to Dynamic Use and Management Elements” in such spaces may reflect the environmental assessment mechanism in Ulrich’s Stress Recovery Theory-the elderly’s vigilant monitoring of potential risk factors (Ulrich, 1983).

In the Multifunctional Areas, the two groups of participants exhibited similar extensive visual scanning behavior but distinctly different emotional responses. This finding may confirm Kaplan’s Attention Restoration Theory: for the elderly with limited cognitive resources, an environment with mixed elements may impose cognitive load, leading to mental fatigue and a low arousal state; for children, however, the same complexity may be perceived as an interesting exploratory challenge (Kaplan, 1995).

Findings from the study on the Green Landscape Areas provide important insights. Although both groups of participants devoted high visual attention to Natural Elements, there were significant intergenerational differences in the stability of their emotional responses. The relatively stable emotional responses of the Child Group support the predictions of the Attention Restoration Theory, indicating that the restorative benefits of natural environments for children are more consistent (Berman, Jonides & Kaplan, 2008). In contrast, the greater variability in the Elderly Group’s emotional responses (with a larger IQR and the most outliers) is associated with Van den Berg’s Perceptual Complexity Theory, which suggests that the restorative effects of natural environments are regulated by multiple factors such as personal experiences, health conditions, and environmental quality (Van den Berg, Joye & Koole, 2016). This finding implies that simple green space area indicators are insufficient to evaluate the benefits of spaces for the elderly group; further attention should be paid to the quality design of natural environments and individualized needs.

### Intergenerational differences in response patterns in social interaction intensity levels

In the High-Intensity Social Interaction Areas, the Child Group exhibits a synergistic pattern of “High Emotional Arousal-High Attention to Amenity and Leisure Facilities”. This indicates that social spaces with clear design and distinct functions can effectively stimulate children’s positive social emotions and interactive behaviors, which is highly consistent with their need for structured play and social interaction during development. In contrast, although the Elderly Group shows higher functional visual attention in such spaces, their emotional arousal level is significantly lower and their responses are more dispersed, presenting a cautious pattern of “Low Emotional Arousal-High Attention to Amenity and Leisure Facilities”. According to the Ecological Model of Aging and the concept of environmental press, older adults are more sensitive to high-intensity and socially demanding environments, particularly when environmental stimulation exceeds their adaptive capacity (Lawton, 1973). As a result, they may adopt a strategy of passive observation rather than active participation to maintain personal space, regulate social rhythm, and avoid cognitive overload or social pressure, a behavioral tendency that has been widely reported in environmental gerontology and aging-related social behavior studies (Gilleard & Higgs, 2010).

The intergenerational differentiation in the Low-Intensity Social Interaction Areas is the most significant, even showing opposition. The Child Group demonstrates “High Emotional Arousal-High Attention to Dynamic Use and Management Elements” here, with the largest number of outliers, indicating that they are active users of such spaces with single functions and simple structure, and regard them as interesting “playgrounds”. However, for the Elderly Group, the Low-Intensity Social Interaction Areas may become a potential “stressor”. They show the highest heterogeneity of emotional responses (the largest number of outliers) here, and combined with their high attention to dynamic elements, this implies a stress pattern of “High Vigilant Monitoring-High Emotional Fluctuation”. This sharp contrast reveals that the same Low-Intensity Social Interaction Areas may be a “paradise” for children and a “stressor” for some elderly people, accurately explaining the neuropsychological root of intergenerational spatial conflicts.

In the Latent Social Interaction Areas, intergenerational differences are reflected in the differences in environmental utilization strategies and benefit stability. The Child Group presents a stable pattern of “Low Emotional Arousal-High Attention to Natural Ecology and Ornamental Elements”, with the most stable emotional responses (the narrowest IQR). This indicates that they mainly use such quiet and natural spaces (*e.g.*, green corners) for low-intensity restorative rest, and the stabilizing effect of natural environments on their emotions is significant and consistent, which supports the predictions of the Attention Restoration Theory (Liu et al., 2024). Although the Elderly Group invests far more visual attention in natural elements than children, showing a pattern of “Extremely High Attention to Natural Ecology and Ornamental Elements —Low Emotional Arousal”, the stability of their emotional responses is significantly lower than that of the Child Group. This suggests that the restorative benefits of natural environments for the elderly group are strongly regulated by factors such as personal health status and environmental quality preferences, and the benefits are not stable.

### Limitations and future research

Despite the contributions of this study, several limitations warrant future investigation. First, while sufficient for exploratory analysis, expanding the sample size and including diverse age cohorts would enhance the statistical robustness and generalizability of the intergenerational framework. Second, the controlled laboratory setting, though ensuring precision, limits ecological validity; future studies should prioritize the deployment of mobile neurophysiological technologies in real-world community environments. Third, moving beyond the current focus on visual perception, integrating multisensory data (*e.g.*, auditory, tactile) would provide a more holistic evaluation of spatial experience. ly, longitudinal research is needed to assess the long-term neurocognitive impacts of design interventions, thereby solidifying the framework’s practical applicability.

## Conclusions

This study established a multimodal research framework integrating computer vision and neuroergonomics to quantitatively investigate the neurocognitive mechanisms underlying intergenerational perceptions of community public spaces. The findings demonstrated fundamental divergences in neuro-behavioral patterns: children exhibit a “synergistic” pattern of high arousal in unstructured play spaces (*e.g.*, Leftover Areas), reflecting an innate preference for exploration. In contrast, the elderly adopt a “cautious” strategy, exhibiting high arousal coupling in Community Service Areas, which indicates a strong psychological reliance on functional amenities and environmental control.

Crucially, Low-Intensity Social Interaction Areas were identified as sites of “spatial tension”—functioning as attractive play environments for children but acting as potential stressors for the elderly. Linear Mixed Model analysis statistically confirmed these divergences, revealing significant interaction effects (Group × Area Typology) and confirming that these differences are rooted in distinct, context-dependent neurobehavioral response patterns rather than mere preference.

To resolve these conflicts, urban design must move beyond universal solutions. We propose a framework focused on three principles: enhancing cognitive affordance in service areas to specifically support elderly independence and reduce cognitive load; implementing “soft boundaries” to legitimize children’s exploratory play while simultaneously safeguarding the elderly’s need for order and safety; and deploying natural elements as “emotional anchors” to physiologically align the baselines of both generations, creating a shared foundation for positive interaction. By respecting these distinct neurocognitive disparities, community spaces can evolve from sites of potential conflict into environments of shared well-being.

##  Supplemental Information

10.7717/peerj.21126/supp-1 Supplemental Information 1 Raw EEG data

## Additional Information and Declarations

### Data Availability

The following information was supplied regarding data availability: The Eye-Tracking Raw Data is available at Zenodo: Lu, D. (2025, November 1). Eye-Tracking Raw Data. Zenodo. https://doi.org/10.5281/zenodo.17500103
The EEG Raw Data is available in the Supplemental File.
